# Engineered pine endophytic *Bacillus toyonensis* with nematocidal and colonization abilities for pine wilt disease control

**DOI:** 10.3389/fmicb.2023.1240984

**Published:** 2023-12-06

**Authors:** Dongzhen Li, Yongxia Li, Xuan Wang, Wei Zhang, Xiaojian Wen, Zhenkai Liu, Yuqian Feng, Xingyao Zhang

**Affiliations:** ^1^Key Laboratory of Forest Protection of National Forestry and Grassland Administration, Ecology and Nature Conservation Institute, Chinese Academy of Forestry, Beijing, China; ^2^Co-Innovation Center for Sustainable Forestry in Southern China, Nanjing Forestry University, Nanjing, China

**Keywords:** pinewood nematode, *Bt*, bacterial colonization, plant-associated microbes, engineering bacteria, *Bacillus*

## Abstract

**Introduction:**

The pinewood nematode (PWN) is responsible for causing pine wilt disease (PWD), which has led to the significant decline of conifer species in Eurasian forests and has become a globally invasive quarantine pest. Manipulating plant-associated microbes to control nematodes is an important strategy for sustainable pest management. However, it has proven difficult to find pine-associated bacteria that possess both nematocidal activity and the ability to colonize pine tissues.

**Methods:**

The stress experiments with turpentine and pine tissue extract were carried out to screen for the desired target strain that could adapt to the internal environment of pine trees. This strain was used to construct an engineered nematocidal strain. Additionally, a fluorescent strain was constructed to determine its dispersal ability in *Pinus massoniana* seedlings through plate separation, PCR detection, and fluorescence microscopy observations. The engineered nematocidal strain was tested in the greenhouse experiment to assess its ability to effectively protect P. massoniana seedlings from nematode infection.

**Results:**

This study isolated a *Bacillus toyonensis* strain Bxy19 from the healthy pine stem, which showed exceptional tolerance in stress experiments. An engineered nematocidal strain Bxy19P3C6 was constructed, which expressed the Cry6Aa crystal protein and exhibited nematocidal activity. The fluorescent strain Bxy19GFP was also constructed and used to test its dispersal ability. It was observed to enter the needles of the seedlings through the stomata and colonize the vascular bundle after being sprayed on the seedlings. The strain was observed to colonize and spread in the tracheid after being injected into the stems. The strain could colonize the seedlings and persist for at least 50 days. Furthermore, the greenhouse experiments indicated that both spraying and injecting the engineered strain Bxy19P3C6 had considerable efficacy against nematode infection.

**Discussion:**

The evidence of the colonization ability and persistence of the strain in pine advances our understanding of the control and prediction of the colonization of exogenously delivered bacteria in pines. This study provides a promising approach for manipulating plant-associated bacteria and using *Bt* protein to control nematodes.

## 1 Introduction

Plant-parasitic nematodes (PPNs) are major pests around the world, causing economic losses. Biological control based on plant-associated microbes is an important process for sustainable pest management, which protects plants against nematodes by inducing plant resistance or targeting nematodes directly and has shown huge promise in applications (Brown, 2018; Liu et al., 2020; Poveda et al., 2020; Topalović et al., 2020). However, there remains a challenge in effectively utilizing and designing plant-associated microbes to achieve long-term colonization and persistence within the plant tissue (Liu et al., 2017; French et al., 2021).

The pinewood nematode (PWN), *Bursaphelenchus xylophilus* (Steiner & Buhrer) Nickle, is a pine-parasitic nematode that invades the wood tissue of pines and feeds on living epithelial cells, resulting in the pine wilt disease (PWD) (Li et al., 2019, 2020; Zhao et al., 2020). As PWN spend the majority of their lives in wood tissue (Son et al., 2010), there is a high demand for beneficial microorganisms to have efficient colonization and dispersal abilities (Nascimento et al., 2015; Wang et al., 2020; Zhang et al., 2020; Wang et al., 2022). The colonization of the nematophagous fungus *Esteya vermicola* in the pine tree xylem has been investigated (Wang et al., 2020, 2022). Compared with fungi, bacteria have a greater abundance and faster growth rate, making them a more promising option for biological control. While many bacteria have been shown to suppress PWN reproduction (Paiva et al., 2013; Huang et al., 2018; Liu Y. et al., 2019; Proença et al., 2019; Zhao et al., 2022), the colonization and spread of these bacteria within pine tissue are still unclear. Thus, a bacterium that can colonize pine tissue and control PWD has yet to be identified.

The genus *Bacillus* is an important component of the plant endophytic microbiome, and *Bacillus thuringiensis* (*Bt*) is a species that produces a wide variety of insecticidal and nematocidal proteins (Melo et al., 2016). *Bt* proteins have been broadly used in agriculture and have not shown significant risk to the environment or human health. Transgenic plants and engineered microbes expressing *Bt* proteins have been designed and commercialized (Sanahuja et al., 2011; Park et al., 2017; Zheng et al., 2018; Pinos et al., 2021; Tilgam et al., 2021). For instance, the engineered bacterium *B. thuringiensis* GO33A, which has broad insecticidal activity against lepidopteran and coleopteran pests, was the first genetically engineered bacterium to be approved for commercialization in China (Wang et al., 2006). Transgenic soybean events expressing the nematocidal protein Cry14Ab are protected from *Heterodera glycines* in both the greenhouse and field (Kahn et al., 2021; McCarville et al., 2023). For PWN, the fungus *Botrytis cinerea* transformed with the *Bt* nematicidal gene Cry5Ba3 has been shown to reduce nematode fitness (Cheng et al., 2018). *Bacillus* spp. with nematocidal activity and colonization ability had not been found, and the engineered bacteria had not been reported. Combining the strategy of engineered bacteria with pine endophytic bacteria may provide new opportunities for controlling pine wilt disease.

In this study, we isolated eight endophytic *Bacillus* strains from pine tissues and examined their ability to adapt to the endogenous environment of the pine tissues. The *B. toyonensis* strain Bxy19, which was isolated from healthy pine stems, was selected to construct engineered strains due to its optimal tolerance against turpentine and pine tissue extract in stress experiments. A fluorescent strain, Bxy19P3GFP, that expresses green fluorescent protein (GFP), was constructed and used to demonstrate colonization and dispersal ability in pine tissue. The engineered strain Bxy19P3C6, which expresses the crystal protein Cry6Aa and demonstrated nematocidal activity, showed remarkable efficacy in controlling nematode infection in greenhouse experiments.

## 2 Materials and methods

### 2.1 Microbial strains, nematodes, and cultivation conditions

*Bacillus* strains used in this study were isolated from different pine tissues (Table 1). The pine samples were sourced from pine forests in Hangzhou, Zhejiang Province, Taian, Shandong Province, and Zhanjiang, Guangdong Province, China. These samples were collected during the period spanning from June to July 2021 and encompassed tree species such as *P. massoniana*, *P. caribaea,* and *P. tabuliformis.* Wood samples were trimmed to no more than 30 mm^3^ in size. The trimmed samples or needles were treated with 75% ethanol for 5 min and then washed thrice with sterile water. The sterilized samples were decorticated and cut into 3 mm pieces. Approximately 2 g of the cut pieces were transferred into 15 mL tubes, to which 5 mL of sterile water was added. The tubes were shaken for 2 h at 28°C. The shaken liquid was spread on nutrient agar (NA). The strains on NA were selected, cultured in nutrient broth (NB), and stored at −80°C. To classify these *Bacillus* strains, the 16S rRNA gene sequences were amplified by PCR with the primers 27F and 1492R (Supplementary Table S1). Each PCR amplification was performed using 2 μL of primers, 25 μL of KOD OneTM PCR Master Mix (TOYOBO, Japan), 21 μL of ddH_2_O, and 1 μL of bacteria liquid as the template. PCR was carried out at 98°C for 5 min and 35 cycles of 98°C for 10 s, 60°C for 5 s, 68°C for 10 s, and then 68°C for 5 min. All PCR products were sent to a commercial sequencing company (BGI, Beijing, China) for PCR purification and sequencing. Sequences were obtained and used for a BLAST search of the EzBioCloud Database to identify the species. Utilizing the top-hit taxon results, we conducted an initial classification of the isolated *Bacillus* strains. The 16S rRNA gene sequences had been submitted to GenBank, and their accession has been presented in Table 1.

**Table 1:** *Bacillus* sp. strains used in this study.

No.	Taxonomic affiliation	Collection places	Pine species	Pine status	Isolated from	Genbank ID
Bxy19	*B. toyonensis*	Hangzhou, Zhejiang	*P. massoniana*	Healthy	xylem	OR770561
Bxy37	*B. toyonensis*	Hangzhou, Zhejiang	*P. massoniana*	Healthy	xylem	OR770562
Bxy158	*B. paramycoides*	Hangzhou, Zhejiang	*P. massoniana*	Diseased	nematode	OR770563
Bxy179	*B. toyonensis*	Hangzhou, Zhejiang	*P. massoniana*	Healthy	root	OR770564
Bxy348	*B. tequilensis*	Zhanjiang, Guangdong	*P. caribaea*	Diseased	needle	OR770565
Bxy396	*B. toyonensis*	Hangzhou, Zhejiang	*P. massoniana*	Healthy	xylem	OR770566
Bxy403	*B. wiedmannii*	Hangzhou, Zhejiang	*P. massoniana*	Diseased	xylem	OR770567
Bxy524	*B. tequilensis*	Taian, Shandong	*P. tabuliformis*	Diseased	branch	OR770568

The PWN nematodes were isolated from pinewood chips infested with *Pinus massoniana* in Ningbo, Zhejiang Province, China, and cultured in our laboratory. Nematodes were cultured on fungal mats of *Botrytis cinerea* grown on potato dextrose agar (PDA) plates at 25°C in the dark.

### 2.2 Stress experiment

**Figure 1:**
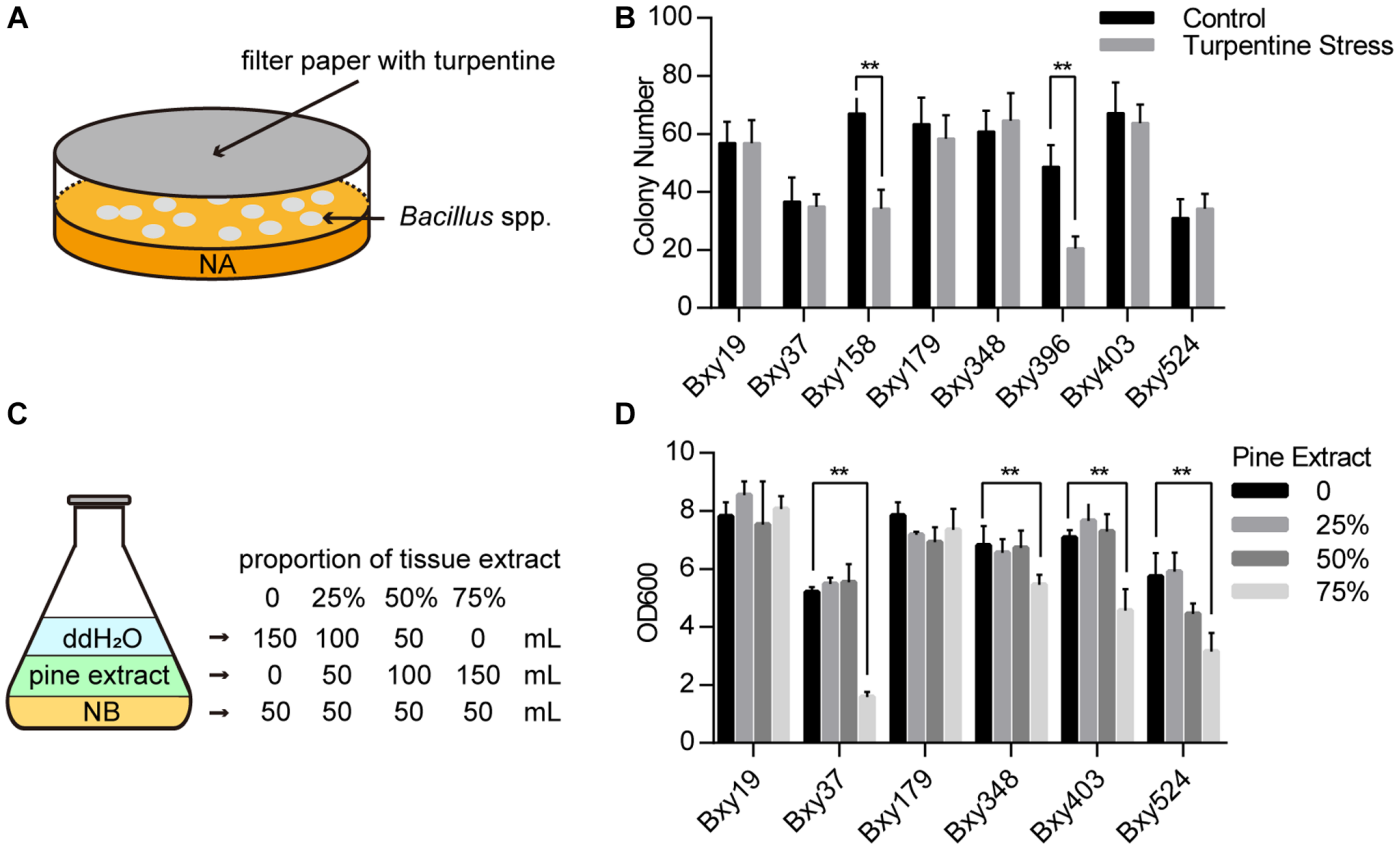
*Bacillus* sp. strain screening for adaptation to turpentine and pine tissue extracts. **(A)** Diagram of a plate stress experiment using turpentine. **(B)** Impact of turpentine on the growth of various *Bacillus* sp. strains in plate stress experiments. **(C)** Diagram of pine tissue extract stress experiment. **(D)** Effects of pine tissue extract on the growth of different *Bacillus* sp. strains. The error bars represent ± SEM. Significant differences between the results were studied using one-way analysis of variance (ANOVA), followed by the Tukey method. “**” indicated *p* < 0.01.

The plate stress experiment was designed to test the bacterial capability of adapting to terpenes. Different *Bacillus* sp. strains were coated onto the NA plate in 9-cm Petri dishes. A piece of sterilized filter paper with 100 μL of terpene liquid was placed inside the cover of the Petri dish (Figure 1A). The Petri dishes were sealed with sealing films and cultured at 28°C. The number of strain colonies on the plates was counted after 24 h. Liquid culture stress experiments were performed to test their capability to adapt to tissue extract. A measure of 50 g needles and 50 g stems were sampled from 3-year-old *P. massoniana.* The tissues were mixed, ground into small pieces, and transferred to a 1,000-mL conical flask. A measure of 500 mL of distilled water was added to the conical flask, which was then shaken at 37°C for 2 h. After mixing, centrifuge the suspension for 1 h, and transfer the supernatant to a new sterilized conical flask. Then, the supernatant was filtered with a 0.22 μm microporous membrane to remove bacteria and fungus. The final product obtained was the pine extract solution. Different amounts of the 0.05 × extract solution were added to the NB culture medium, and then water was added to reach a final volume of 200 mL (Figure 1C). Inoculate the *Bacillus* sp. strain into the mixed culture medium and incubate at 28°C for 10 h. Then, determine the absorbance value of the bacterial solution at OD600 to assess the growth status of the strain. These tests were performed with three replicates.

### 2.3 Construction of engineered strains

The plasmid vector pP43NMK-P43-sfGFP used in this study was purchased from Hangzhou Fenghai Biotechnology Co., Ltd. (Hangzhou, China). In order to express the Cry protein constitutively in *B. toyonensis*, we utilized the *cry3A* gene promoter (Pcry3a) (García-Gómez et al., 2013) for protein expression. Pcry3a promoter could work at the vegetative growth stage in *B. thuringiensis,* which can increase the stability of the ribosome-transcript complex and enhance the synthesis of Cry protein (Wang et al., 2006). We synthesized the promoter sequence at Nanjing GenScript Biotech Corporation (Nanjing, China). To replace the P43 promoter of pP43NMK-P43-sfGFP with Pcry3a, the Pcry3a sequence with 16 bp homologous sequences with the target insertion site on the vector pP43NMK-P43-sfGFP was cloned using the primers P3-F and P3-R by PCR (Supplementary Table S1), which was used for the homologous recombination reaction. Meanwhile, the vector pP43NMK-P43-sfGFP was amplified and linearized by PCR with the primers P43NMK-F and P43NMK-R. The PCR products of the linearized vector and Pcry3a sequence were linked by ClonExpress Ultra One Step Cloning Kit V2 (Vazyme, Nanjing, China). The recombined plasmids were transformed into *Escherichia coli* DH5α and sent to the company for sequencing. Successfully constructed strains and recombined plasmids are selected, preserved, and used for subsequent experiments. We further replaced the ampicillin-resistance gene on the plasmid with the kanamycin-resistance gene using the same method. The kanamycin-resistance gene was synthesized and cloned using the primers Kan-F and Kan-R (Supplementary Table S1). The recombinant plasmid was amplified and linearized by PCR with the primers P43NMK-F2 and P43NMK-R2 (Supplementary Table S1). The PCR products of the linearized vector and the kanamycin-resistance gene were linked using the above-mentioned method. The recombinant plasmid pP43NMK-P3-GFP with Pcry3a and kanamycin-resistance genes was obtained. Then, the recombinant plasmid pP43NMK-P3-GFP was electroporated following the methods used in the past (Lereclus et al., 1989). The fluorescent bacterial strains are observed under a fluorescence microscope (Axio Imager A2, Zeiss, Oberkochen, Germany) to confirm the success of constructing the fluorescent bacterial strains.

Similarly, we replaced the *gfp* gene with the *cry6Aa* gene to obtain the recombinant plasmid pP43NMK-P3-Cry6Aa. The vector pP43NMK-P3-GFP was amplified and linearized by PCR with the primers P3NMK-F and P3NMK-R (Supplementary Table S1). The *Cry6Aa* gene was synthesized and cloned using the primers Cry6A-F and Cry6A-R. The PCR products of the linearized vector and *cry6Aa* gene were linked. The recombinant plasmid pP43NMK-P3-Cry6Aa with the *cry6Aa* gene was obtained. Then, the recombinant plasmid pP43NMK-P3-Cry6Aa was electroporated into Bxy19 to obtain the engineered strain Bxy19P3C6. For the identification of the engineered strain Bxy19P3C6, crystalline protein staining and SDS-PAGE (sodium dodecyl sulfate-polyacrylamide gel electrophoresis) were carried out. Bacterial isolates were cultured in NB at 28°C for 5 days. After incubation, a bacterial smear was prepared, air-dried, and fixed by gentle heating. The bacterial smear was flooded with methanol for 30 s, followed by the addition of 0.5% basic fuchsin. The sides were heated gently until steam appeared. Finally, slides were rinsed with water and visualized under the microscope for the presence of oval toxin crystals. Meanwhile, 10 mL of fermentation liquor from different strains was collected and mixed with 1 mL of cell lysis buffer (20 mM Na_2_CO_3_, 10 mM EDTA, 0.1 mM DTT, pH = 10), which was lysed at 37°C for 3 days. A measure of 30 μm lysate was mixed with SDS-PAGE loading buffer and boiled for SDS-PAGE.

### 2.4 Nematocidal activity of engineered strain Bxy19P3C6

Bacterial isolates were cultured in NB at 28°C for 24 h. The bacterial solution was collected by centrifugation and washed three times with a sterile PBS solution (potassium phosphate buffer). The bacterial solution was then diluted to 1 × 10^4^ cells/mL for nematocidal experiments. Nematodes were collected and washed three times with a sterile PBS solution. To remove symbiotic microorganisms on the surfaces of the nematodes, they were immersed in a 15% H_2_O_2_ solution for 10 min and washed three times with a sterile PBS solution. Subsequently, 200 μL of the diluted bacterial solution was added to each well of a 40-well plate, and 500 nematodes were added to each well. The 48-well plate was covered with sealing film and cultured in darkness at 25°C. After 24 h, the mortality rate of nematodes was calculated.

### 2.5 Colonization of fluorescent strain Bxy19P3GFP

A measure of 200 mL of fluorescent strain Bxy19P3GFP fermentation liquor (1 × 10^8^ cells/mL) was sprayed onto the surface of 3-year-old *P. massoniana* seedlings using an alcohol spray bottle in the greenhouse, which was named the sprayed group. A measure of 100 μL of fermentation liquor (1 × 10^8^ cells/mL) was injected into the stems of other seedlings, which was named the injected group. At the lower part of the stem, we used a 0.5-millimeter-diameter drill bit to create a hole at a downward angle, injected 100 μL of fermentation liquor into the hole using a pipette, and sealed the hole by wrapping it with sealing film. For the control group, the trees were sprayed and injected with NB liquid. Each group included 10 seedlings. The fresh tissues were sampled at 10, 30, and 50 days after inoculation for subsequent assessment.

A modified protocol (Bodenhausen et al., 2013) was used to separate epiphytic and endophytic microbes. For the samples in the sprayed group, the leaf and stem material were weighed, and 10 mL of 0.1 M PBS with a pH of 8.0 was added to tubes for each gram of the sample. The tubes were sonicated for 1 min and vortexed for 10 s; this process was repeated twice. Wash steps were also repeated once. The wash for the leaves was filtered using a 0.22 μm millipore filter membrane (Sangon, Shanghai, China). The filter membrane was placed into a 2-mL centrifuge tube containing 1 mL of PBS. After sonication for 1 min and vortexed for 10 s, a bacterial suspension was obtained. The suspension was then subjected to gradient dilution and plated for culture. The remaining suspension was centrifuged at 12,000 rpm for 10 min to extract DNA for PCR detection. The sonicated sample was weighed, cut into pieces, and pulverized in 10 mL of PBS for each gram of the sample. After sonication for 1 min and vortexed for 30 s, the supernatant was collected by centrifugation at 5000 rpm for 10 min and then subjected to gradient dilution and plated for culture. The remaining supernatant was used for DNA extraction and PCR detection.

For the samples in the injected group, stem samples around the injection hole were sampled and separated into two parts: one part was used for plate separation and PCR detection; the other part was used for the fluorescence microscopy observations of the colonization of strains in the host pine tree stems. The stem samples were weighed, cut into pieces, and pulverized in 10 mL of PBS for each gram of the sample. The pulverized stem samples were sonicated, vortexed, and collected according to the above method. The supernatants were obtained and used for spread plate and PCR detection.

Meanwhile, the needles and stem samples were assessed by sectioning and fluorescence microscopy to observe the colonization of the fluorescent strain Bxy19P3GFP. The surface of the needle was gently washed with sterile water to remove dust. Fresh white radishes were cut into pieces, each about 3 cm long, 2 cm wide, and 1 cm thick. The function of the white radish pieces were to hold the needles, making it easier for the slicer to secure the needles. The cleaned needles are placed between two slices of white radish to secure them. Then they were placed on a slicer (HuaSu, Jinhua, China) to make fresh tissue sections by transverse cutting. At the same time, a razor blade was used to make longitudinal sections of the needle samples. For plant stem tissues, after similarly rinsing the surface dust with clean water, it is placed on a slicer for making fresh sections by transverse, longitudinal, and tangential cuts. The obtained sections are observed under a fluorescence microscope.

### 2.6 DNA extraction and sequence amplification

The genomic DNA of all the samples was extracted using the TIANamp Bacteria DNA Kit (TIANGEN, Beijing, China). The sfGFP-F and sfGFP-R primer pairs (Supplementary Table S1) were used to amplify the sfGFP gene in different samples to detect the colonization of fluorescent strain Bxy19P3GFP. PCR reactions were carried out with 15 μL of Phusion® High-Fidelity PCR Master Mix (New England Biolabs, USA), 0.2 μM of forward and reverse primers, and approximately 20 ng template DNA. Thermal cycling consisted of initial denaturation at 98°C for 1 min, followed by 30 cycles of denaturation at 98°C for 10 s, annealing at 50°C for 30 s, and elongation at 72°C for a different time that was adjusted according to the length of the fragment to amplify. Finally, there was a final elongation step of 72°C for 5 min. All PCR products were sent to a commercial sequencing company (BGI, Beijing, China) for PCR purification and sequencing.

### 2.7 Disease control by engineered strain Bxy19P3C6

Engineered strain Bxy19P3C6 fermentation liquor (1 × 10^8^ cells/mL) was prepared for the greenhouse experiment. Three-year-old *P. massoniana* seedlings with similar intraspecific heights were grown in a greenhouse at 25 with 80% RH. One month before the greenhouse experiments, 90 *P. massoniana* seedlings were divided into three groups. One treatment included 30 seedlings that were injected with the fermentation liquor (50 μL) at the lower parts of the stems and inoculated with 5,000 nematodes by artificial injection after 10 days, namely the injected group. One treatment included 30 seedlings that were sprayed with the fermentation liquor (200 mL) and inoculated with 5,000 nematodes by artificial injection after 10 days, namely the injected group. The pathogenic treatment included 30 seedlings that were sprayed with the Bxy19 fermentation liquor (200 mL) and injected with the fermentation liquor (50 μL) at the lower parts of the stems and inoculated with 5,000 nematodes by artificial injection after 10 days, namely the control group. When inoculating nematodes, we also used a 0.5-millimeter-diameter drill bit to create a hole at a downward angle at the lower part of the stem, injected 50 μL of fermentation liquor into the hole using a pipette, and sealed the hole by wrapping it with sealing film. All the treatments were observed constantly until the leaves were wilted and the seedings died, which was approximately 60 days. Then the mortality was counted.

### 2.8 Statistical analyses

All of the experiments were performed with three replicates. Significant differences were studied using one-way analysis of variance (ANOVA), followed by the Tukey method in SPSS (v20.0; SPSS Inc., Chicago, IL, USA). The level of significance in all cases was 95% (*p*-value <0.05). In the greenhouse experiment, significance analysis was mainly performed using the chi-square test. During the chi-square test, Pearson’s chi-square test was used for significance analysis.

All statistical analyses were visualized with GraphPad (v7.0.).

## 3 Results

### 3.1 Screening of *Bacillus* sp. strains for the construction of engineered strains

Pine tissues contain abundant oleoresins and metabolites. In particular, terpene compounds were biosynthesized and accumulated in large quantities when PWN infected pine (Liu et al., 2021). These defense chemicals not only prevent nematode infection but also have unpredictable effects on endophytic bacteria. So, the desirable biocontrol bacteria should adapt to this complex environment, preferably. Eight endophytic *Bacillus* strains were isolated from the diseased and healthy pines (Table 1).

We tested their capability of adapting to terpenes through plate stress experiments with turpentine, whose main components were terpenes (Figure 1A). The result that the numbers of six strain colonies on the plates were not influenced by turpentine indicated these strain growths were not inhibited and showed terpene stress tolerance (Figure 1B). Furthermore, by adding aseptic pine tissue extract with different volumes to the NB nutrient solutions with the same volumes, we test their capability of adapting to the condition in the pines (Figure 1C). If these strains were only cultured in tissue extracts without NB nutrient solutions, the population quantity of all strains did not increase and even decreased when the incubation time was too long. When tissue extracts were 25 and 50%, the reproduction of all strains in NB mixed with tissue extract showed no difference with the NB mixed with the same volume of water. When tissue extracts were 75%, only Bxy19 and Bxy179 stained appeared to be unaffected (Figure 1D). In contrast to Bxy179, which was isolated from pine roots, Bxy19 was isolated from a healthy pine stem. Bxy19 was selected for the construction of the engineered strain because it was characterized by tolerance to turpentine and pine tissue extracts.

### 3.2 Construction of engineered strains

**Figure 2:**
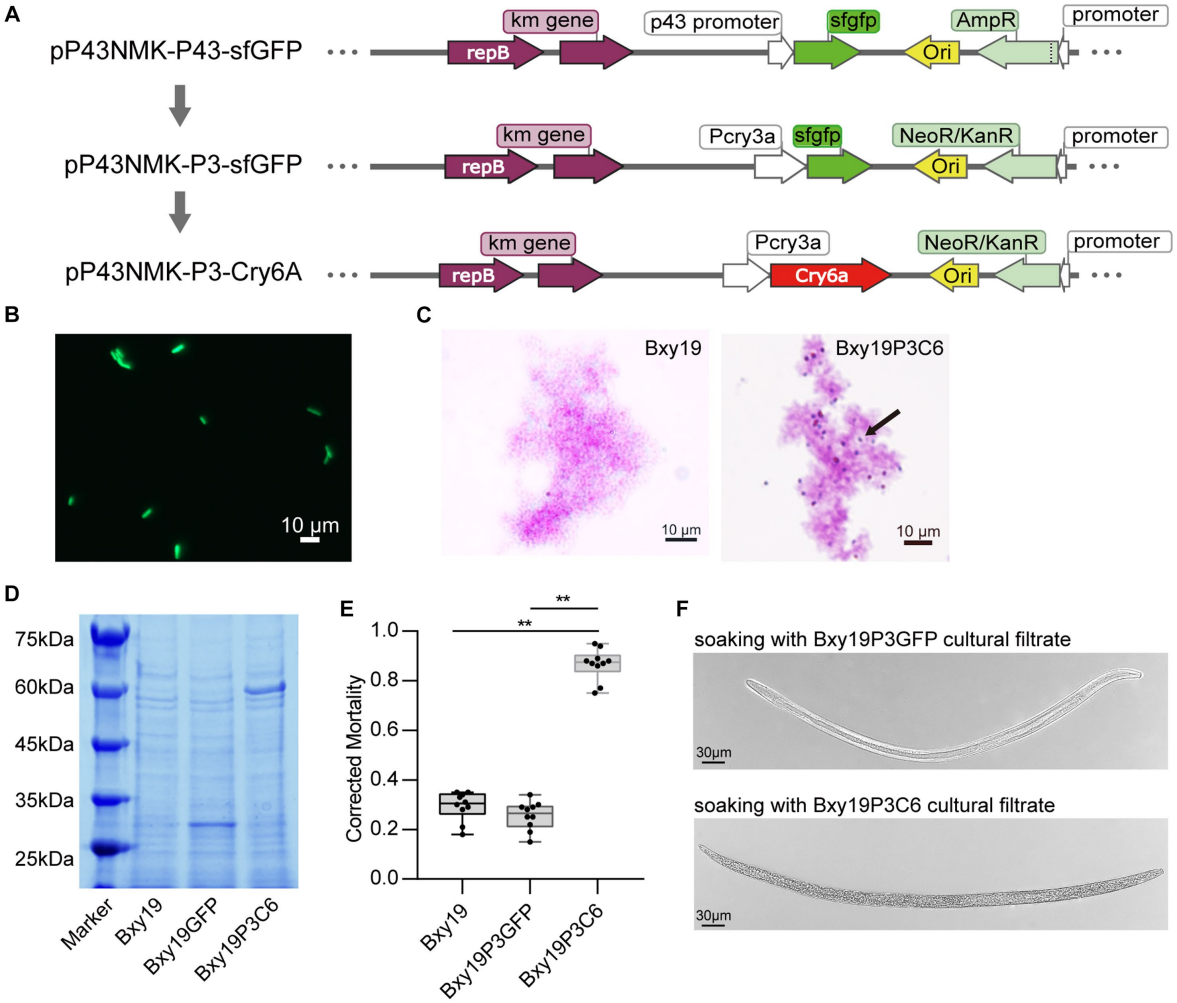
Construction of engineered *Bt* strains. **(A)** Diagram of the construction of a recombinant plasmid. **(B)** Fluorescence microscope observation of fluorescent strain Bxy19P3GFP. Scale bars were 10 μm. **(C)** Crystal protein staining for the detection of the engineered strain Bxy19P3C6. Arrows show the presence of oval toxin crystals at either end of cells. Scale bars were 10 μm. **(D)** Detection of crystal protein from Bxy19P3C6 using SDS-PAGE. **(E)** The nematicidal activity of the engineered strains Bxy19P3GFP and Bxy19P3C6. The error bars represent ± SEM. Significant differences between the results were studied using one-way analysis of variance (ANOVA), followed by the Tukey method. “**” indicated *p* < 0.01. **(F)** Morphological changes in nematodes treated with Bxy19P3C6 strains. Scale bars were 30 μm.

To express the heterologous protein in the Bxy19 strain, we constructed the recombinant plasmids pP43NMK-P3-GFP and pP43NMK-P3-Cry6Aa (Figure 2A). Finally, the engineered strains Bxy19P3GFP and Bxy19P3C6 were obtained by the transformation of pP43NMK-P3-GFP and pP43NMK-P3-Cry6Aa into the recipient Bxy19 strains, respectively. Strong green fluorescence was observed in the Bxy19P3GFP strain (Figure 2B), which indicated the recombinant plasmid pP43NMK-P3 was suitable for Bxy19 and acted with strong promoter activity. For Bxy19P3C6, oval toxin crystals were presented at either end of cells by carbol-fuchsin stain (Figure 2C). SDS-PAGE analysis showed that the engineered strain Bxy19P3C6 produced 54 kDa crystal protein and Bxy19P3GFP produced 27 kDa GFP protein (Figure 2D).

Furthermore, the nematicidal activity of these strains was measured by soaking the nematodes in the filter liquor of fermentation broth. Bxy19P3C6 was engineered to express the Cry6Aa protein. Cry6Aa is known to have nematocidal activity and has been previously shown to be effective against pinewood nematodes. It likely acts as a pore-forming toxin that disrupts the intestinal cells of nematodes after being ingested (Peng et al., 2011; Huang et al., 2016). After treatment with the different strains for 24 h, the corrected mortality of the engineered strain Bxy19P3C6 against nematodes was >80%, which was significantly different from Bxy19 and Bxy19P3GFP (Figure 2E). The typical infection symptoms were that the integrity of the intestine was severely disrupted and intestinal cells appeared to lyse (Figure 2F), which led to bradykinesia, body stiffness, and death. This indicated that the nematocidal activity of Bxy19P3C6 is attributed to the expression of the Cry6a protein.

Meanwhile, we test the bacterial viability of the engineered strains. The growth curves indicated that the Bxy19 and Bxy19P3GFP strains had similar viability. However, Bxy19P3C6 strains grew more slowly and cracked more rapidly (Supplementary Figure S1A). When the strains lived up to 50 generations, the heredity stability measure showed that 65% of Bxy19P3C6 strains contained recombinant plasmid, which was lower than Bxy19P3GFP (82%) (Supplementary Figure S1B). So, it seemed that expressing the Cry6a crystal protein had some negative effects on the Bxy19P3C6 strains. Subsequently, the fluorescent strain Bxy19P3GFP was used to study the colonization in pine tissues, and the engineered strain Bxy19P3C6 was used to verify its control efficiency against PWD.

### 3.3 Colonization of pine tissues by fluorescence strain Bxy19P3GFP

**Figure 3:**
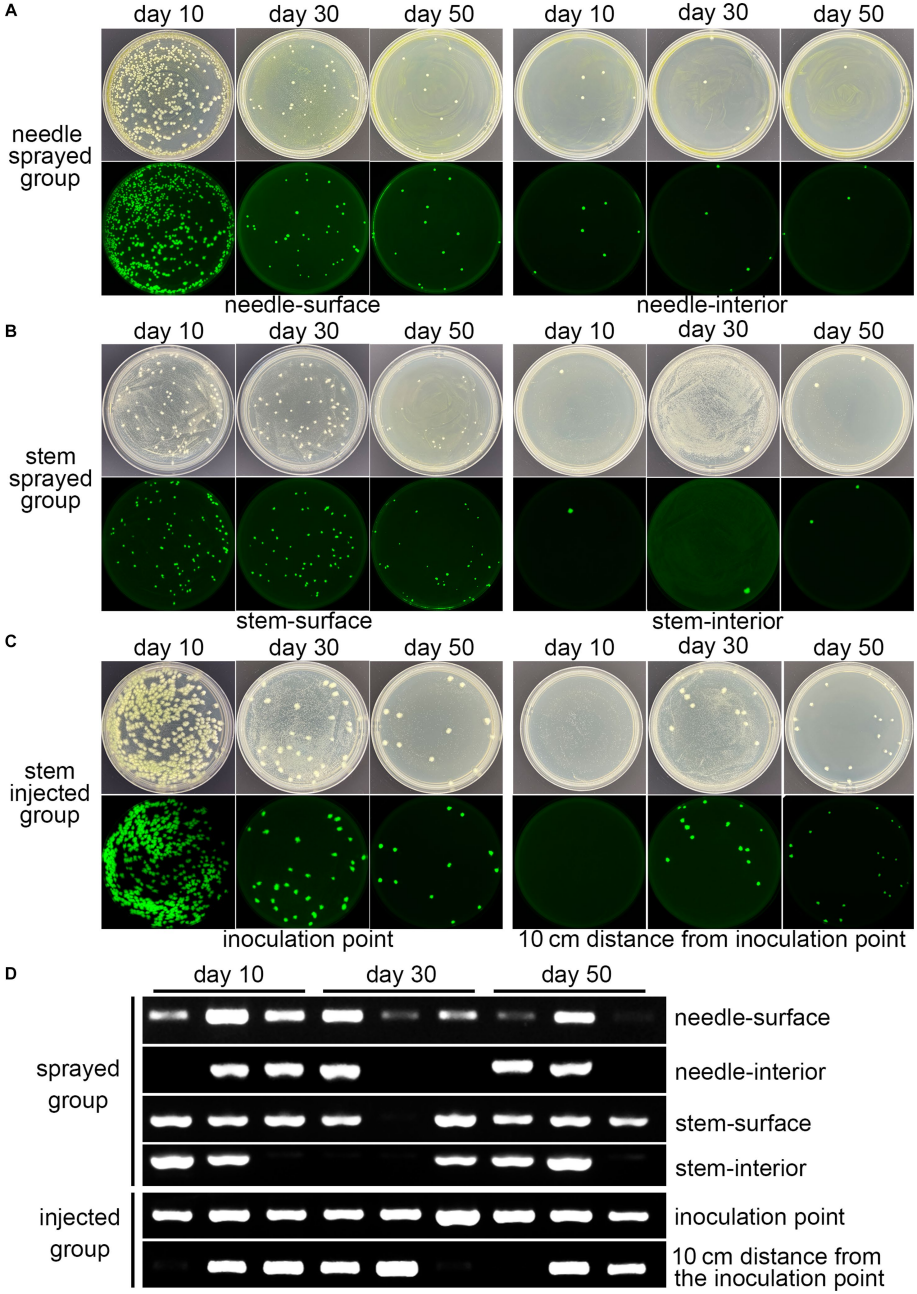
Detection of the colonization of fluorescent strain Bxy19P3GFP. Plate isolation **(A–C)** and PCR detection **(D)** of Bxy19P3GFP strains from different pine tissues in the spraying group and injection group at different time points. The images above were bright-field images, and the images below were GFP-fluorescence-field images.

Spray and trunk injection were the usual inoculation methods. The fluorescent strain fermentation liquor was sprayed onto the surface of 3-year-old *P. massoniana* seedlings and injected into the stems, respectively, named the sprayed group and the injected group. Fresh tissues were sampled for plate separation, PCR detection, and fluorescence microscopy observations on days 10, 30, and 50. Abundant Bxy19P3GFP strains could be isolated from the surface of the needle and stem in the sprayed group during the experiments (Figures 3A,B). The population quantity decreased from day 10 to day 50. A few strains were also isolated from the interior of needles and stems in the sprayed group (Figures 3A,B). For the injected group, abundant fluorescent strains were also isolated from the stem tissues near the inoculation point, which also decreased from day 10 to day 50 (Figure 3C). Furthermore, we detected whether the fluorescent strains spread in the stems. There were no fluorescent strains isolated from the stem tissues within 10 cm of the inoculation point on day 10, and the fluorescent strains were successfully isolated and seemed to increase in amount on days 30 and 50 (Figure 3C). PCR detection of the sfGFP gene also showed similar results (Figure 3D). Stable PCR signals could be detected in most of the samples, including the needle surface, stem surface, and inoculation point. In addition, PCR signals could also be detected in partial samples at every sampling time point, including the needle interior, stem interior, and stem (10 cm from the inoculation point). More importantly, the results demonstrated that the fluorescent strain Bxy19P3GFP appeared to spread to the internal tissues from the surface and inoculation point and could survive for at least 50 days.

**Figure 4:**
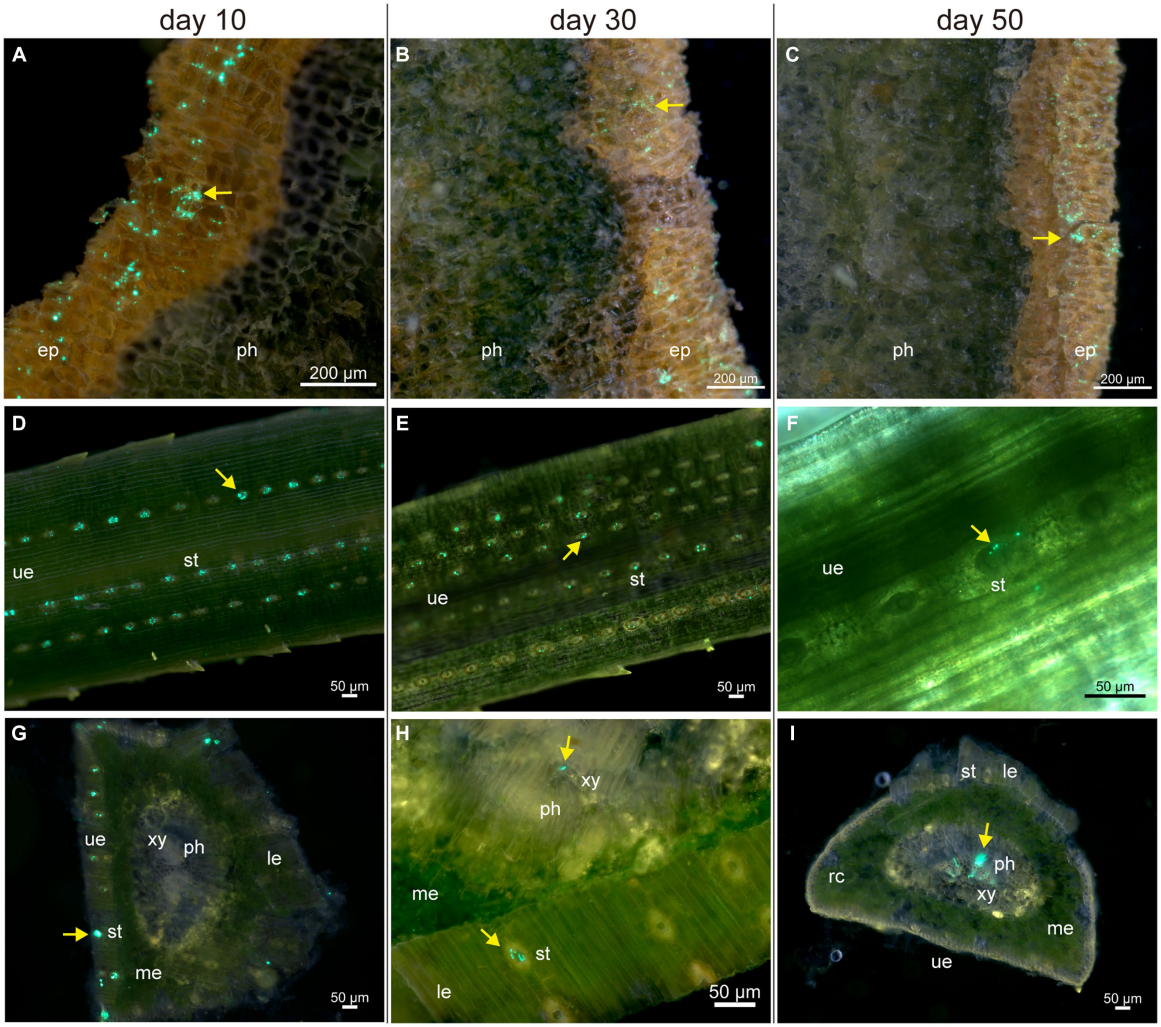
Fluorescence microscope analyses of the colonization of pine tissues by Bxy19P3GFP in the spraying group. The fluorescent strain Bxy19P3GFP was observed on the surface of the epidermis **(A–C)**, needles **(D–F)**, and inner parts of needles **(G–I)** on days 10, 30, and 50. Yellow narrows indicate the fluorescent signal of Bxy19P3GFP. Abbreviations used in the figure are: ep (epidermis), le (lower epidermis), me (mesophyll), ph (phloem), rc (resin canal), st (stomata), ue (upper epidermis), and xy (xylem). Scale bars of picture a–c were 200 μm. Scale bars of picture d–i were 50 μm. The bright field images are shown in Supplementary Figure S2.

For a detailed description of pine tissue colonization by Bxy19P3GFP, we prepared fresh tissue sections for fluorescence microscopy observation. After spraying Bxy19P3GFP fermentation liquor, many motile Bxy19P3GFP cells were detected on the surface of the epidermis during the whole experimental period, and no colonization of the interior of stems was observed (Figures 4A–C). It seemed that Bxy19P3GFP could not cross the periderm without wounds and other ostioles. Meanwhile, large amounts of Bxy19P3GFP cells were observed to cumulate in the vicinity of needle stomates, which also decreased from day 10 to day 50 (Figures 4D–F). No extensive colonization of the interior of needles was observed on days 10 and 30 (Figures 4G,H). Although rare Bxy19P3GFP cells were also observed in the interior of needles, we cannot rule out the possibility that these cells were brought from the surface when the fresh needle was sliced. However, obvious fluorescence signals were observed in the vascular bundle on day 50 (Figure 4I). We also observed that Bxy19P3GFP cells were clustered at some sites instead of distributed uniformly along the vascular bundle. Microscopy inspection revealed that sprayed Bxy19P3GFP gained access to the interior of needle tissue via stomata and later established colonies in the vascular bundle.

**Figure 5:**
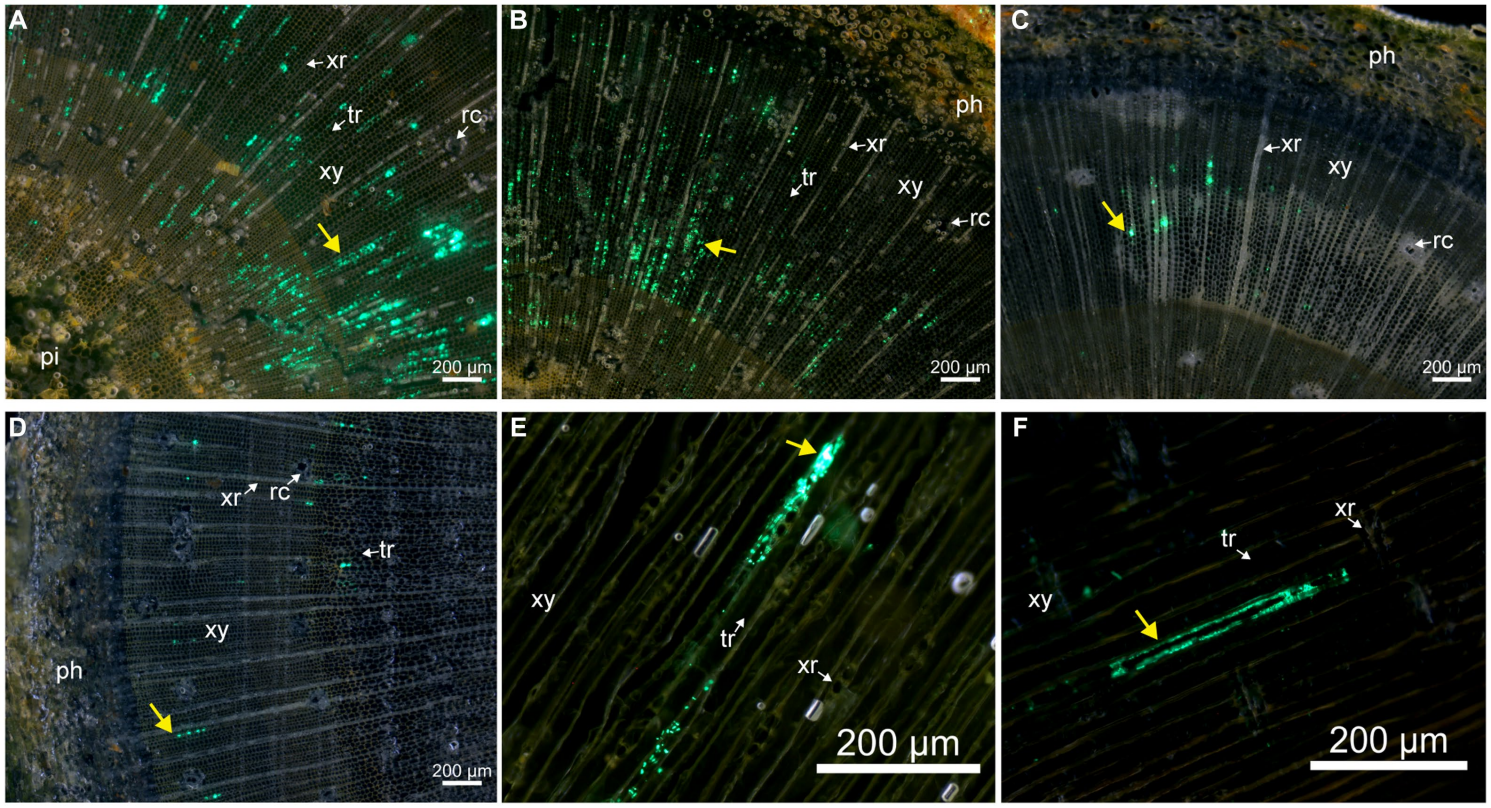
Fluorescence microscope analyses of the colonization of pine stem by Bxy19P3GFP in the injection group on day 50. The fluorescent strain Bxy19P3GFP was observed in stem transverse sections that were located 0.5 **(A)**, 1 **(B)**, 1.5 **(C)**, and 2 **(D)** cm from the inoculation points, and also in stem radial sections **(E)** and tangential sections **(F)** that were located 1 cm from the inoculation points. Yellow narrows indicate the fluorescent signal of Bxy19P3GFP. Abbreviations used in the figure were: ph (phloem), pi (pith), rc (resin canal), tr (tracheid), xy (xylem), and xr (xylem ray). Scale bars were 200 μm. The bright field images are shown in Supplementary Figure S3.

In the injected group, numerous fluorescent signals could always be observed near the inoculation points up to day 50. We further observed the stem sections at different distances from the inoculation points to explore the bacterial spread on day 50. No evident and credible fluorescent signal was observed in the phloem and pith. An obvious fluorescent signal was mainly observed in the xylem tracheid. Some tracheids were filled with fluorescent signals approximately 0.5 and 1 cm farther from inoculation points, which were distributed radially along the xylem ray direction on the stem transection (Figures 5A,B). Fewer fluorescent signals were observed in the tracheid, approximately 1.5 and 2 cm farther from inoculation points. Fluorescent cells could also be observed clearly on the tracheid wall (Figures 5C,D), which indicated that Bxy19P3GFP strains spread in the xylem tracheid. We also observed the stem radial section (Figure 5E) and tangential section (Figure 5F), which presented that Bxy19P3GFP strains colonized in some xylem tracheid and adhered to the wall.

### 3.4 Disease control by engineered strain Bxy19P3C6

**Figure 6:**
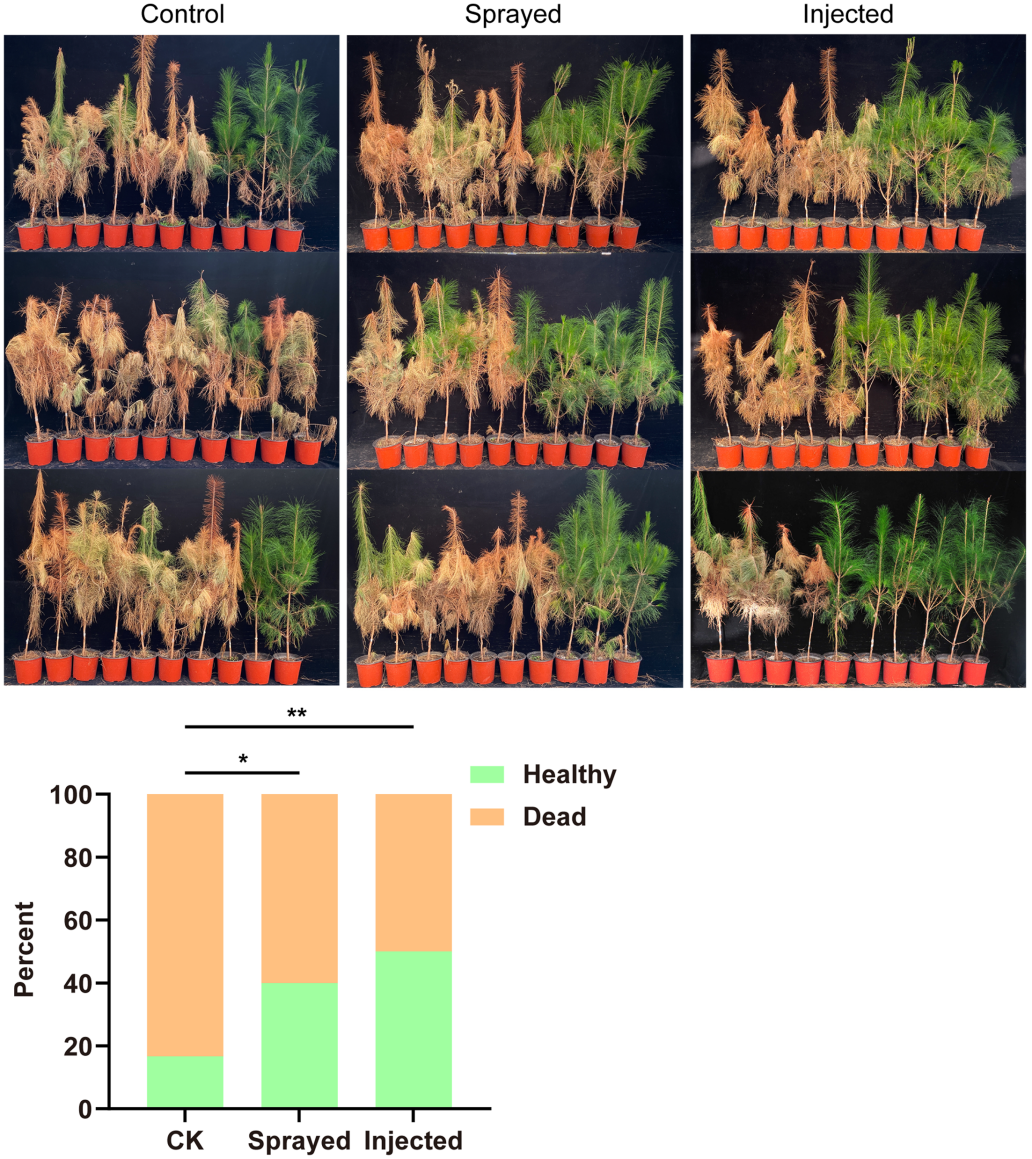
Experimental test of Bxy19P3C6 that protected plants from nematode infection. This experiment had three treatments, namely the control group (sprayed and injected Bxy19 and infected by nematodes), sprayed group (sprayed Bxy19P3C6 and infected by nematode), and the injected group (injected Bxy19P3C6 and infected by nematode). Each column of images shows the disease status of 30 pine seedlings in each treatment group. The number of dead and healthy seedlings was counted and used for the chi-square test, with results presented at the bottom. Compared between the control group and sprayed group: χ^2^_pearson_ (1) = 4.02, *p* = 0.045, showing a significant difference marked with “*.” Similarly, compared between the control group and the injected group: χ^2^_pearson_ (1) =7.50, *p* = 0.006, showing a significant difference marked with “**.” There was no significant difference between the injected group and the sprayed group.

The engineered strain Bxy19P3C6, which expressed nematocidal Cry6Aa proteins, was conducted in the greenhouse experiment to test if it could effectively protect 3-year-old *P. massoniana* seedling plants from nematode infection (Figure 6). The results showed that the control group lost 25 pine seedlings, with a mortality rate of 83%. The sprayed group lost 18 pine seedlings, with a mortality rate of 60%. The injected group lost 15 pine seedlings, with a mortality rate of 50%. After a chi-square test, the injection group and the spraying group showed significant differences from the control group. The healthy pine seedlings did not show abnormalities, which indicated treatment with Bxy19P3C6 fermentation liquor had no negative influence on seeding growth. The greenhouse experimental results showed that both spraying and injecting the engineered strain Bxy19P3C6 had considerable efficacy against nematode infection.

## 4 Discussion

Controlling pine wilt disease using microbes associated with pine trees is a sustainable and environmentally friendly approach. However, the colonization ability of biocontrol microbes within pine trees has been a limiting factor for this strategy. In this study, a *B. toyonensis* strain, Bxy19, adapted to the internal environment of pine trees, was screened and transformed into an engineered nematocidal strain, Bxy19P3C6. This new strain had colonization ability in pine trees and displayed effective control against pine wood nematodes.

Screening desirable target strains was critical to the engineering of bacterium construction. High levels of terpene and phenol substances in pine tissues usually make it difficult for the microbes isolated from other environments, such as soil and gramineous plants, to adapt (Chen et al., 2006; Keeling and Bohlmann, 2006). Especially when infected with pests and pathogens, the content of terpene substances and resins usually increases notably (Kuroda, 1991; Fukuda, 1997; Hara et al., 2006; Bohlmann, 2012). Although these substances play an important role in plant defense responses, they also have unpredictable influences on the endophytic microbial community and usually have a negative impact on the colonization of exogenously delivered strains (Wu et al., 2013; Zhang et al., 2021, 2022). Our study screened for more adaptable strains through stress experiments with turpentine and pine tissue extract. Although all the candidate strains were isolated from pine tissues, not all of them were able to adapt to the experimental conditions, which may be an important reason for affecting the abundance of strains in plants. However, relying solely on this method for screening target strains may not be sufficient, as there are other intricate factors, such as the microbial ecosystem and metabolic flow within the plant, that also play a role in determining the colonization of strains. There was a need to research more effective and accurate screening methods.

Detection of the colonization and spread of strains within plants was beneficial to predicting control efficiency and evaluating safety (Parke and Gurian-Sherman, 2001). Unlike the extensive studies on grass plants (Czajkowski et al., 2010; Compant et al., 2011; Hannula et al., 2021; Synek et al., 2021), the colonization of strains in woody plants was relatively understudied. Our study confirmed that the fluorescent strain Bxy19GFP could enter needles via stomata and colonize the vascular bundle of the needles. Whether these strains enter the stem were still unclear. Although no fluorescent strains were observed inside the stem after spraying, they could be detected by PCR and plate isolation experiments. We speculated that the strain cannot directly penetrate the periderm of the stem and that some wounds or other ostioles on the stem may be important routes of entry. After injection, fluorescent strains could colonize and spread in the tracheid. The nematocidal crystal proteins could be transported to whole pines through the tracheid, which may provide effective protection for plants.

Control of the nematode by using the strain should be divided into direct and indirect control. In this study, Bxy19P3C6 was an engineered strain obtained through genetic modification of the wild-type strain Bxy19. The direct mechanism of Bxy19P3C6 is to kill nematodes by expressing the nematocidal crystal protein Cry6Aa. Through *in vitro* soaking experiments, we were able to confirm that the strain Bxy19P3C6 possesses nematocidal activity, whereas the wild-type strain Bxy19 and the fluorescent strain Bxy19P3GFP, which did not express Cry6a protein, did not exhibit nematocidal activity. The wild-type strain Bxy19 was sprayed on the seeding surface and injected into seedling stems in the control group and health group. Current studies have shown that treatment with the wild-type strain Bxy19 did not negatively affect pine seedlings. The wild-type strain Bxy19 also did not have any protective effect. The engineered strain Bxy19P3C6 was derived from the wild-type strain Bxy19. Apart from expressing Cry6Aa, it could not significantly impact other characteristics of the strain, such as enzyme activity, toxin production, or antibiotic secretion. Therefore, the effectiveness of the engineered strain Bxy19P3C6 in controlling pine wilt disease was primarily attributed to the Cry6Aa protein. Perhaps the Bxy19P3C6 strain had some other benefits for pine trees, but these effects were not sufficient to help pine trees resist pine wilt disease. The long-term effects and impacts on the endogenous microbiome of pines remain to be investigated.

Engineered bacterial strains constructed in the laboratory usually lack the satisfactory adaptive ability to complex natural environments (Sheth et al., 2016; Liu Y. X. et al., 2019). First, the growth activity of strains may be affected by genetic transformation. Whether it produced other types of crystal proteins is still unclear. To minimize the negative effect on the strain, we used the promoter and crystal protein identified from *Bt* for genetic transformation. Even so, expressing Cry6Aa still resulted in a significant decrease in the activity of the strain. Second, a stable endophytic microbial community had been established in the plant. The microbial communities exist in complex, dynamic consortia with highly interconnected networks of metabolic and ecological interactions that have yet to be unraveled (Carlström et al., 2019; Voorhees et al., 2020; Debray et al., 2022). Thus, it is difficult to control or predict the long-term colonization of exogenously delivered bacteria. Even if the introduced strains can achieve stable colonization, they may outcompete endogenous commensals and adversely alter the balance of the microbial ecosystem. In our study, the application of the engineered strain Bxy19P3C6 did not appear to have an adverse impact on pine health. Their population quantity decreased continuously after inoculation and eventually disappeared, which was beneficial for environmental security. However, whether short-term colonization could help achieve sustainable pest management requires more experiments in the field.

This study sheds new light on the use of *Bt* proteins to control pine wilt disease. Pinewood nematodes could feed on both pine parenchyma cells and fungal cells by inserting style into cells and sucking up cell contents (Futai, 2013). *Bt* crystal protein typically killed nematodes by disrupting intestinal cells when the crystal proteins entered the nematode’s intestinal tract (Zhang et al., 2016). Hence, transgenic pines and fungi were the appropriate delivery vectors. Transgenic crops expressing crystal proteins have been extensively studied and applied to the control of root-knot nematodes and cyst nematodes (Li et al., 2007; Kahn et al., 2021). The fungi that expressed crystal proteins could also reduce the population of pine wood nematodes (Cheng et al., 2018). However, the techniques and strategy of pine-resistance transgenesis were not yet mature, and the breeding cycle of pine was too long. Fungi were less abundant than bacteria in pines. In this study, *Bt* crystal protein was expressed in bacteria, which showed a desired control efficiency in the greenhouse. The control effect of the injected group was better than that of the sprayed group, which we speculated may be due to the lower abundance of the sprayed strains in the pine tissues. Although the pine wood nematode did not feed on bacteria, *Bt* protein can be released into the environment along with the release of spores and has the potential to enter the nematode’s intestine. This study suggested that using bacteria expressing *Bt* protein was a promising approach for controlling pine wilt disease.

It is not clear the introduction of genetically modified microorganisms (GMMs) in current practices (Cravens et al., 2019). The release of GMMs into the environment may raise biosafety concerns related to their potential ecological impacts. First, a major concern is the unintentional transfer of recombinant plasmids to other organisms. Transgenic microorganisms may contain genes, such as antibiotic-resistance genes, which could spread in the environment, potentially leading to antibiotic-resistance issues or other ecological impacts. In our study, we used the kanamycin-resistance gene because it is widely used in transgenic plants. In future research, we will consider the exclusion of antibiotic-resistance genes just like the engineered bacterium *B. thuringiensis* GO33A, which was the first genetically engineered bacterium to be approved for commercialization in China (Wang et al., 2006). The second question is whether the use of engineered bacteria could lead to drug resistance in nematodes. In theory, the long-term heavy use of *Bt* proteins does carry the risk of target nematodes developing resistance, even leading to non-target organisms developing resistance. Third, the impact of GMMs on the symbiotic microorganisms of soil and plants is challenging to assess. The long-term impacts require more investigation. In this study, the population quantity of the engineered strain Bxy19P3C6 decreased continuously after inoculation and eventually disappeared, which may be beneficial for environmental security. However, caution is still required regarding the release of this strain into the environment. On the one hand, further adjustments to this engineered microorganism are needed. On the other hand, long-term environmental safety assessments should be carried out.

In this study, we screened a *Bacillus* strain with a strong ability to colonize and survive in the internal environment of pine trees through stress experiments. The strain was transformed to express the *Bt* nematocide protein and used as a biological control agent against pine wilt disease. First, we have described in detail for the first time the colonization and diffusion of the bacteria in the needles and stems of the pine tree, indicating that the inoculation of strains within the tree can be achieved through spraying and injection, which is important for controlling forest pests and diseases through aerial protection methods. Second, when there is insufficient understanding of the plant endophytic microbiome and metabolic network, using the top-down approach that uses carefully selected environmental variables (such as turpentine in our study) to screen candidate microorganisms is an important method for utilizing endophytic microorganisms. Third, the expression of *Bt* nematocide protein by endophytic bacteria is a feasible alternative, and it is also an important approach for utilizing *Bt* protein and plant-associated microbes.

## Data availability statement

The data presented in the study are deposited in the Figshare, accession number: https://doi.org/10.6084/m9.figshare.24631404.v1.

## Author contributions

YL and DL designed and supervised the project. XWa, WZ, XWe, ZL, and YF collected all pine samples and bacteria strains. DL performed all experiments and all statistical analyses. XZ and DL obtained funding. All authors revised and approved the manuscript.
