# Supplementary material for: Engineered pine endophytic Bacillus toyonensis with nematocidal and colonization abilities for pine wilt disease control
Source: Front Microbiol. 2023 Dec 6;14:1240984. doi: 10.3389/fmicb.2023.1240984 (PMC10731049; doi:10.3389/fmicb.2023.1240984)
Supplement: Supplementary file 1 [file Data_Sheet_1.docx]

Supplementary Material

Engineered Pine Endophytic *Bacillus toyonensis* with Nematocidal and Colonization Abilities for Pine Wilt Disease Control

Dongzhen Li^1,2^, Yongxia Li^1,2,*^, Xuan Wang^1,^2, Wei Zhang^1,2^, Xiaojian Wen^1,2^, Zhenkai Liu^1,2^, Yuqian Feng^1,2^, Xingyao Zhang^1,2^

*** Correspondence:** Yongxia Li: liyongxiaxjs@163.com

## 1 Supplementary Figures

**
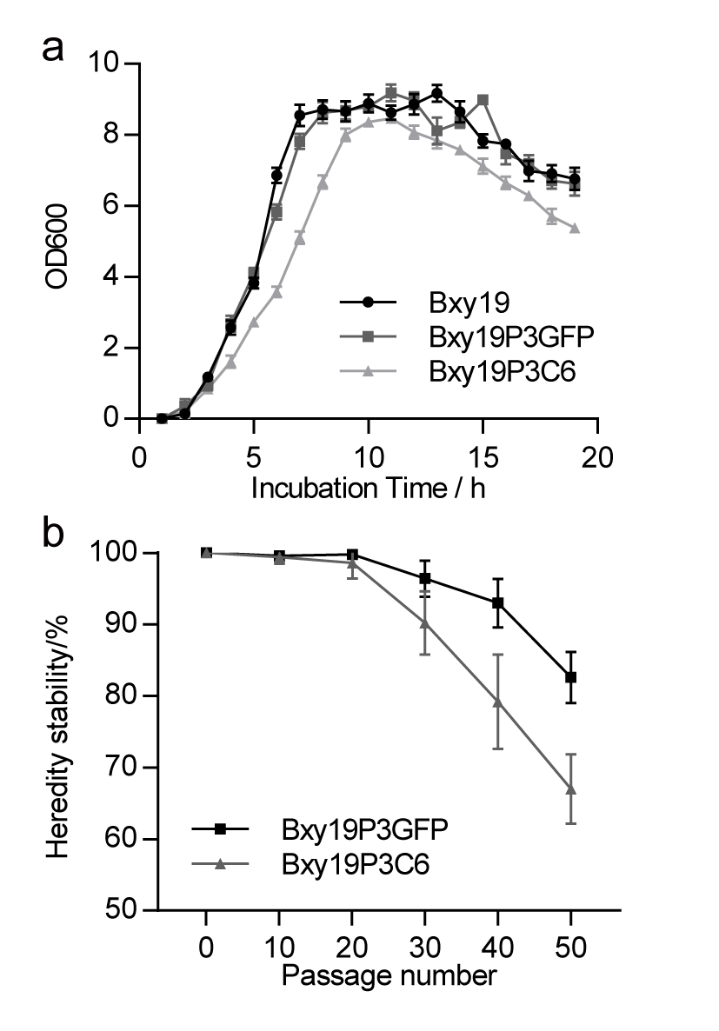
**

**Supplementary Figure S1.** **The growth curves of wild-type strain Bxy19, engineered strain Bxy19P3GFP and Bxy19P3C6 (a) and the heredity stabilities of engineered strain (b).**


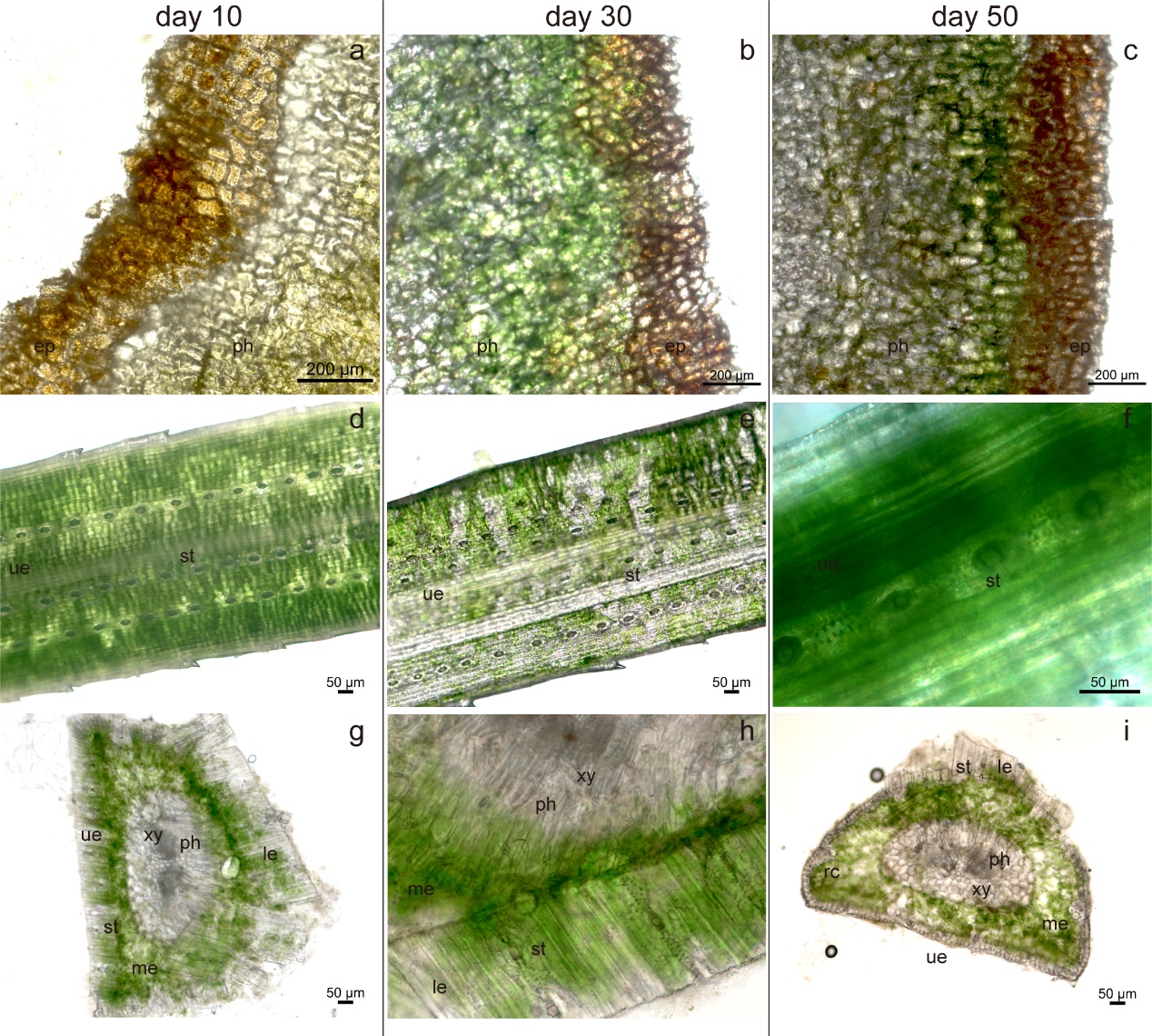


**Supplementary Figure S2** **The bright field images of Figure 4.** The fluorescent strain Bxy19P3GFP was observed on the surface of the epidermis (a-c), needles (d-f), and inner parts of needles (g-i) on day 10, 30 and 50. Yellow narrows indicated the fluorescent signal of Bxy19P3GFP. Abbreviations used in the figure are: ep (epidermis), le (lower epidermis), me(mesophyll), ph (phloem), rc(resin canal), st(stomata), ue(upper epidermis), xy (xylem). Scale bars of picture a-c were 200μm. Scale bars of picture d-i were 50 μm.


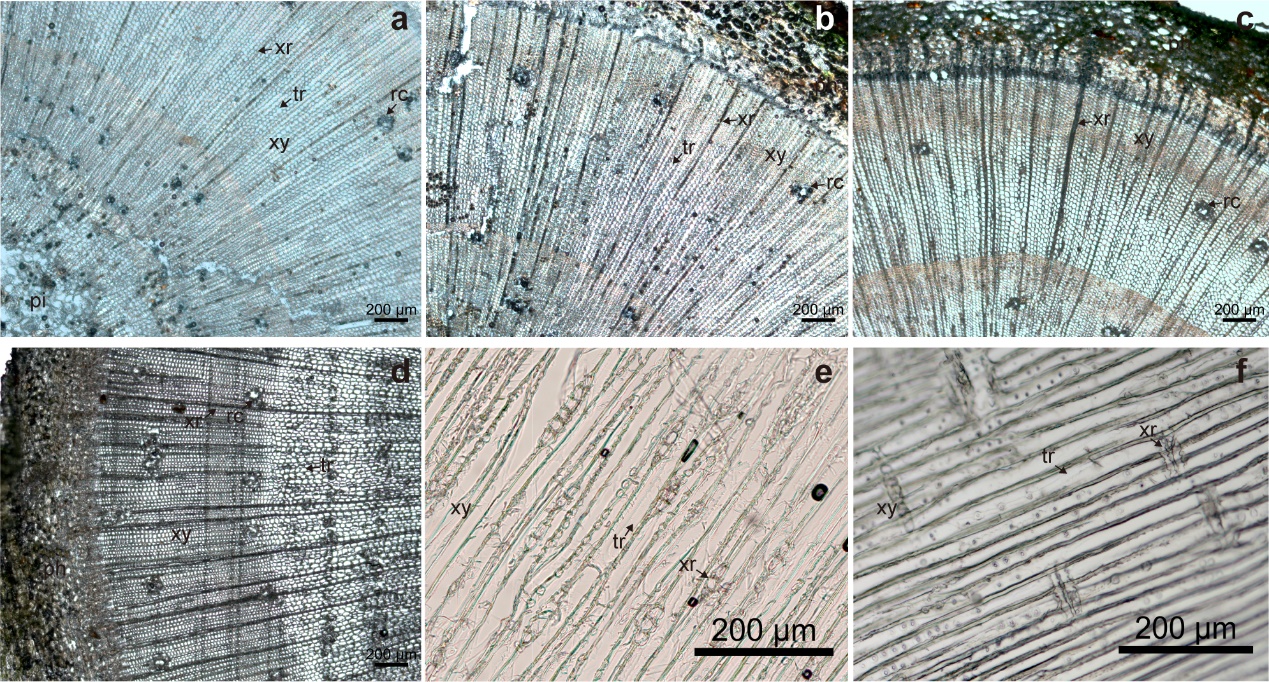


**Supplementary Figure S3 The bright field images of Figure 5.** The fluorescent strain Bxy19P3GFP was observed in stem transverse sections that were located 0.5 (a), 1 (b), 1.5 (c), and 2 (d) cm from the inoculation points, and also was observed in stem radial section (e) and tangential section (f) that were located 1 cm from the inoculation points. Abbreviations used in the figure were: ph (phloem), pi (pith), rc (resin canal), tr (tracheid), xy (xylem), xr (xylem ray). Scale bars were 200 μm.

**Supplementary Table S1 List of primers**

| Primer name | Sequence (5′-3′) |
| --- | --- |
| P3-F | ttgagcaactggatccagcttaattaaagataatatctttgaattg |
| P3-R | cttctcctttgctcatttttcttcctccctttcttatcataataca |
| P43NMK-F | tatctttaattaagctggatccagttgctcaaaaaaatctcggtca |
| P43NMK-R | aaagggaggaagaaaaatgagcaaaggagaagaacttttcactgga |
| Kan-F | aaacttggtctgacagtcagaagaactcgtcaagaaggcgatagaa |
| Kan-R | ttgaaaaaggaagagtatgattgaacaagatggattgcacgcaggt |
| P43NMK-F2 | tgacgagttcttctgactgtcagaccaagtttactcatatatactt |
| P43NMK-R2 | catcttgttcaatcatactcttcctttttcaatattattgaagcat |
| Cry6A-F | aaagggaggaagaaaaatgattattgatagtaaaacgactttacct |
| Cry6A-R | gattacgccaagcttcttaattattataccaatccgaattattata |
| P3NMK-F | tactatcaataatcatttttcttcctccctttcttatcataataca |
| P3NMK-R | ttggtataataattaagaagcttggcgtaatcatggtcatagctgt |
| 27F | agagtttgatcctggctcag |
| 1492R | ggttaccttgttacgactt |
| sfGFP-F | atgagcaaaggagaagaacttttcactggagttgt |
| sfGFP-F | tatttgtagagctcatccatgtgtaatcccagcag |
